# Predictive value of serum ALT and T-cell receptor beta variable chain for HBeAg seroconversion in chronic hepatitis B patients during tenofovir treatment

**DOI:** 10.1097/MD.0000000000006242

**Published:** 2017-03-10

**Authors:** Jiezuan Yang, Dong Yan, Renyong Guo, Jiajia Chen, Yongtao Li, Jun Fan, Xuyan Fu, Xinsheng Yao, Hongyan Diao, Lanjuan Li

**Affiliations:** a State Key Laboratory for Diagnosis and Treatment of Infectious Diseases, Collaborative Innovation Center for Diagnosis and Treatment of Infectious Diseases, The First Affiliated Hospital, College of Medicine, Zhejiang University, Hangzhou; b Department of Laboratory Medicine, The First Affiliated Hospital, College of Medicine, Zhejiang University, Hangzhou; c Department of immunology, Zunyi Medical Univesity, Zunyi, China.

**Keywords:** antiviral therapy, chronic hepatitis B, gene melting spectral pattern, HBeAg seroconversion, predictive biomarker

## Abstract

Effective antiviral therapy plays a key role in slowing the progression of chronic hepatitis B (CHB). Identification of serum indices, including hepatitis B e antigen (HBeAg) expression and seroconversion, will facilitate evaluation of the efficacy of antiviral therapy in HBeAg-positive CHB patients. The biochemical, serological, virological parameters, and the frequency of circulating CD4^+^CD25^+^ regulatory T cell (Treg) in 32 patients were measured at baseline and every 12 weeks during 96 weeks of tenofovir disoproxil fumarate (TDF) treatment. The relationship between the hepatitis B virus (HBV) deoxyribonucleic acid (DNA) and Treg and alanine aminotransferase (ALT) levels was analyzed, respectively. The molecular profiles of T-cell receptor beta variable chain (TRBV) were determined using gene melting spectral pattern. For the seroconverted 12 patients, ALT declined to normal levels by week 24 and remained at this level in subsequent treatment; moreover, the predictive cutoff value of ALT for HBeAg seroconversion (SC) was 41.5 U/L at week 24. The positive correlation between HBV DNA and Treg and ALT was significant in SC patients, but not in non-SC patients. Six TRBV families (BV3, BV11, BV12, BV14, BV20, and BV24) were predominantly expressed in SC patients at baseline. The decline of ALT could be used to predict HBeAg seroconversion for CHB patients during TDF treatment. In addition, the profile of Tregs and TRBVs may be associated with HBeAg seroconversion and could also be a potential indicator for predicting HBeAg SC and treatment outcome for CHB patients.

## Abstract 2

Supplemental Digital Content is available in the text

## 1 Introduction

More than 350 million people worldwide are chronically infected with hepatitis B virus (HBV), and chronic hepatitis B (CHB) is one of the most common infectious diseases in Southeast Asia. HBV infection is a major cause of chronic liver disease, including cirrhosis, liver failure, and hepatocellular carcinoma (HCC).^[1]^ It is a generally accepted viewpoint that antiviral therapy against HBV plays a pivotal role in determining the outcomes of CHB and preventing HCC. An effective antiviral therapy for CHB can control viral replication, promote serum alanine aminotransferase (ALT) normalization, loss of hepatitis B e antigen (HBeAg) expression, seroconversion (SC) for HBeAg, loss of hepatitis B surface antigen (HBsAg) expression, and seroconversion for HBsAg. This is achieved in only a small number of patients.^[2,3]^ Moreover, long-term suppression of HBV can lead to regression of fibrosis and cirrhosis in patients with chronic HBV infection.^[4,5]^

HBV infection shows no direct cytopathology in infected hepatocytes, both hepatic cell damage and viral control are immune-mediated.^[6,7]^ Considering HBeAg-positive CHB patients, HBeAg SC is a satisfactory therapeutic goal after antiviral treatment, which is associated with the restoration of the immune capabilities of the host and the quantity and function of regulatory T cells (Tregs),^[8,9]^ especially with restoration of the immune response of HBV-specific T lymphocytes to HBV.^[8,10]^ In addition, T-cell receptor (TR) is the characteristic molecule on the T-cell membrane, and its expression profile could indicate the nature of the immune response in the host.^[11]^

In our previous study, we found that the gene melting spectral pattern (GMSP) analysis for TR beta variable chain (TRBV) could help to evaluate the various clinical stages of chronic HBV infection.^[12,13]^ In addition, using GMSP, we discovered that TRBV11, BV15, and BV20 families might be relevant to the favorable outcomes of subjects who recovered from acute HBV infection^[14]^; however, the characteristics of TRBV in peripheral lymphocytes from SC or non-SC HBeAg-positive CHB patients during tenofovir disoproxil fumarate (TDF) treatment is very little known. Moreover, the pattern of ALT level and Treg frequency in SC or non-SC patients with TDF treatment is not well described. These parameters could be used as predictors of seroconversion and successful treatment in HBeAg-positive CHB following antiviral treatment.^[13,15]^

In this study, the kinetic patterns of biochemical, virological, and CD4^+^CD25^+^ Treg in CHB during TDF treatment were investigated. The predictive value of ALT levels in CHB patients undergoing HBeAg SC was analyzed, and the profile of characteristic TRBV was also determined. The dynamic relationship between ALT levels and Treg frequency or HBV deoxyribonucleic acid (DNA) load in patients undergoing HBeAg SC or not were longitudinally analyzed.

## 2 Materials and methods

### 2.1 Subjects

Thirty-five consecutive outpatients with HBeAg-positive CHB were enrolled at the Department of Infectious Disease, the First Affiliated Hospital, College of Medicine, Zhejiang University between November 2011 and September 2012. All patients were positive HBsAg for at least 6 months before treatment, naive with oral TDF (300 mg/d), and their more detail inclusion and exclusion criteria were presented in our and others’ reports.^[16–18]^ In addition, the patients with diabetes, severe systemic illness, or HCC, or who were pregnant or breast-feeding or receiving immunosuppressive (immunoregulatory) therapy were excluded. The study conformed to the ethical guidelines of the Declaration of Helsinki. Informed consent was obtained from all patients. The First Affiliated Hospital, College of Medicine, Zhejiang University medical ethics committee approved all procedures involving human subjects.

HBeAg SC is defined as the loss of HBeAg expression in the peripheral blood of the host (quantitative HBeAg < 1.00 S/CO, signal/cutoff ratio), together with the antibodies that recognize HBeAg (anti-HBeAg) (quantitative hepatitis B e antibody < 1.00 S/CO), the detailed HBeAg measurements are shown (Tables 1 and 2, Supplemental Digital Content 1, 2). Finally, only 32 CHB patients who had completed 96 weeks of antiviral treatment were enrolled and analyzed in the study (3 withdrew, including 1 who withdrew consent, 2 whose protocol deviated). Subjects were classified into HBeAg SC (n = 12) or non-HBeAg SC (n = 20) groups, depending on whether they had undergone HBeAg SC by week 72 of TDF treatment (no HBeAg seroconversion was found during the following treatment). Healthy controls (n = 20) with normal serum ALT level and undetectable HBsAg were recruited from the Health Examination Center of the First Affiliated Hospital of Zhejiang University and were matched for age and gender with CHB patients. The baseline clinical parameters are shown in Table 1.

**Table 1:**
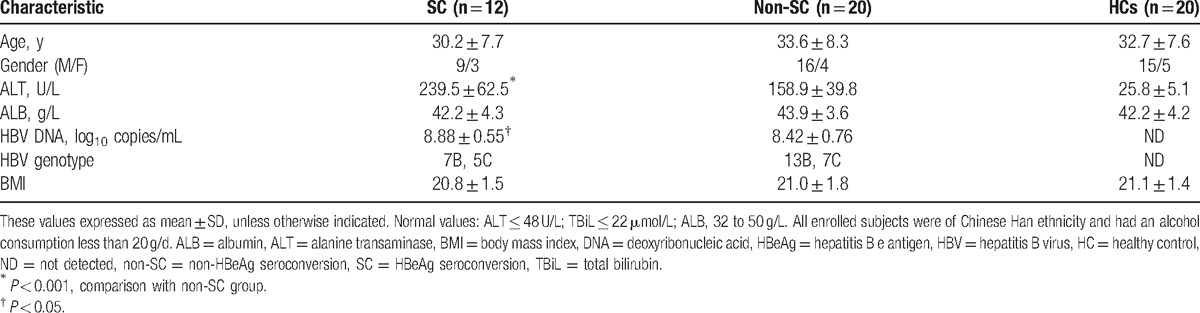
Baseline clinical demographics of subjects enrolled.

**Table 2:**
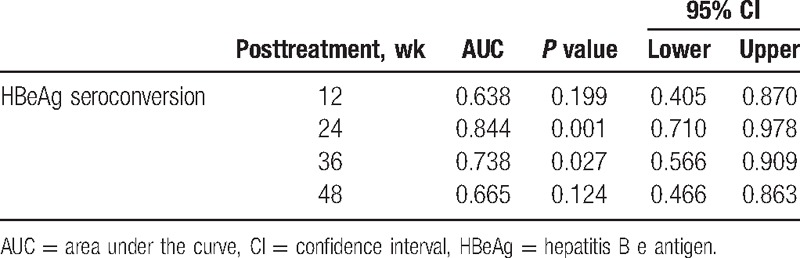
Predictive value of changing ALT level for HBeAg seroconversion.

### 2.2 Assessment of biochemical, serological, and virological parameters

Patients visited the outpatient clinic at least every 12 weeks for routine examinations and laboratory assessments. Routine liver biochemistry and HBV serological and virological parameters were determined at the onset of the study for baseline data and at these 12-week follow-up visits. The seromarkers were determined in the central laboratory of the First Affiliated Hospital, College of Medicine, Zhejiang University. The detail detection methods were provided in our previous study.^[17]^

### 2.3 Separation of peripheral blood mononuclear cell and flow cytometry analysis

Peripheral blood mononuclear cells (PBMCs) were isolated using Ficoll-Paque (STEMCELL Technologies Inc., Vancouver, Canada) density gradient separation from >10 mL peripheral fresh whole blood treated with EDTA K2 anticoagulant. The percentage of the CD4^+^CD25^high^ Treg population in CD4^+^ T cells was determined using flow cytometry analysis as described previously.^[18]^ In addition, intracellular staining of FoxP3 was conducted using fluorescently labeled antibody anti-CD3, anti-CD4, and anti-CD25 for surface maker staining, followed by fluorescein isothiocyanate (FITC)-anti-FoxP3 (eBiosciences, San Diego, CA) staining after permeabilization. Other fluorochrome-conjugated Abs-specific surface markers, including peridinin-chlorophyll-protein-anti-human leukocyte antigen - antigen D related, FITC-anti-CD45RA, and allophycocyanin-anit-CD45RO, were used to confirm the CD4^+^CD25^+^ Tregs as described in our previous study.^[13]^

### 2.4 Total RNA extract and cDNA synthesis

Total ribonucleic acid (RNA) was extracted from PBMCs using an SV Total RNA Isolation System (Promega, Madison, WI) according to the manufacturer's instructions and was immediately reverse-transcribed to complementary (c)DNA using a RevertAid First Strand cDNA Synthesis Kit (MBI Fermentas, Waltham, Massachusetts, USA). The more detailed protocols were provided in our previous study.^[12]^

### 2.5 Analysis of GMSP for TRBV family

GoTaq qPCR Master Mix (Promega, Madison, WI) was used for real-time polymerase chain reaction (PCR) of GMSP analysis. The reaction volume consisted of 25-μL Master Mix containing 50 to 150 ng cDNA as a template to generate GMSPs, as previously described.^[19]^ Following PCR-product melting curve analysis, the peak shape pattern of the melting curve for the 24 TRBV gene families was determined by plotting the inverse of the first derivative of the reduction in the fluorescence signal (−dF/dT) as a function of temperature, and the GMSPs of the TRBV families were determined as previously described.^[19]^

### 2.6 Identification of skewed TRBV family

Skewed TRBV gene families were identified by GMSP profile and classified into 3 kinds: oligoclonal, monoclonal, and multiclonal expansion (not skewed). The details are described in our previous publications.^[14,18]^

### 2.7 Sequencing monoclonal TRBV family

The GMSP profile of a TRBV gene family showing a single peak was selected for cloning and sequencing to determine the degree of homogeneity within the complementary district region 3 (CDR3). This approach was described in our previous report.^[20]^ The TRBV gene sequences that were obtained were translated into the corresponding amino acid sequence using Chromas (version 2.22; Technelysium, South Brisbane, QLD, Australia), and basic local alignment search tool (http://blast.ncbi.nlm.nih.gov/Blast.cgi) was used to define the CDR3 and BJ segments in each TRBV family.

### 2.8 Statistical analysis

All data were analyzed using SPSS version 19.0 (SPSS Inc., Chicago, IL). Continuous variables with skewed distributions were analyzed using the Mann–Whitney *U* test or Kruskal–Wallis test. Paired-related data were analyzed using the Wilcoxon paired test. The correlation between 2 parameters was performed using Spearman bivariate correlation. Categorical variables were analyzed using a χ^2^ test or Fisher exact test. A receiver operating characteristic (ROC) curve was performed to identify the optimal cutoff value of the ALT levels for the prediction of the HBeAg seroconversion. The area under the curve (AUC) of the ROC curve was calculated for these variables, and AUC values closer to 1 indicated a higher predictive capacity of response to therapy. The *P* < 0.05 was considered statistically significant.

## 3 Results

In the present study, no other liver-related complications occurred during treatment, and no serious adverse effects or hepatitis flare were observed. In addition, no patient underwent clearance of HBsAg during 96 weeks of TDF treatment.

### 3.1 Potential predictive value of ALT level for HBeAg seroconversion

**Figure 1:**
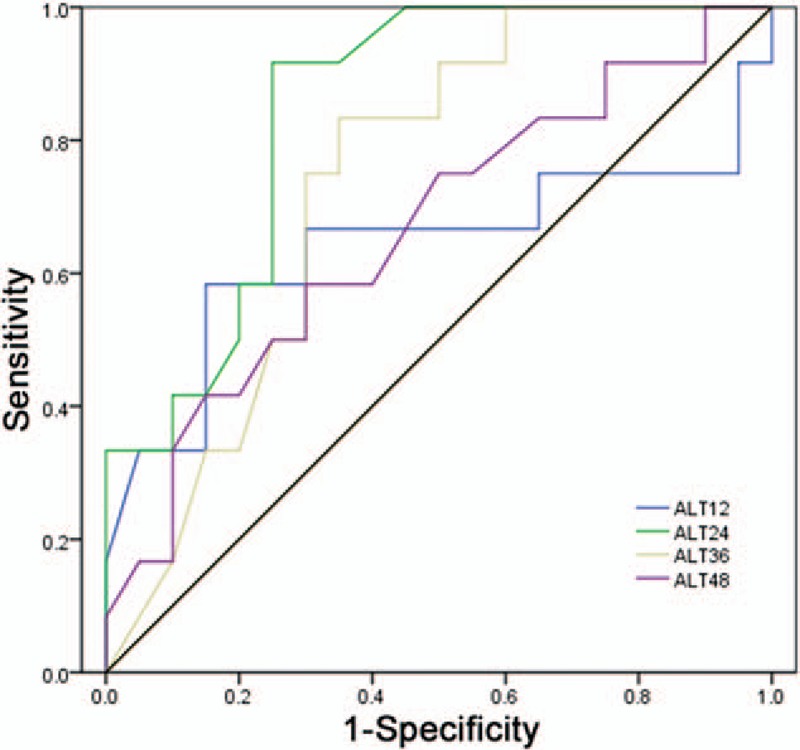
Receiver operating characteristic curves between hepatitis B e antigen seroconversion (SC) and non-SC patients with tenofovir disoproxil fumarate treatment by the change in alanine aminotransferase levels at weeks 12, 24, 36, and 48. ALT = alanine transferase, non-SC = nonhepatitis B e antigen seroconversion, ROC = receiver operating characteristic, SC = hepatitis B e antigen seroconversion, TDF = tenofovir disoproxil fumarate.

ROC curve analysis was used to assess the discrimination in the performance of the changing ALT level for the prediction of patient responses to TDF therapy. The AUC was calculated for each parameter (Table 2), and the optimal cutoff value, the sensitivity, and specificity for prediction were determined in ROC curves (Fig. 1). In terms of HBeAg seroconversion of CHB patients, the AUC was 0.638 at week 12, 0.844 at week 24, 0.738 at week 36, and 0.665 at week 48. With respect to the ALT level, the optimal cutoff value for the prediction of the patient with HBeAg SC was 41.5 IU/L (sensitivity = 91.7% and specificity = 75.0%) at week 24; moreover, the positive and negative predictive values are 68.8% and 93.8%, respectively. However, at week 36, the cutoff value was 31.5 IU/L (sensitivity = 83.3% and specificity = 65%), with the positive and negative predictive values 58.8% and 86.7%, respectively.

### 3.2 Normalization of ALT level between SC and non-SC CHB patients

**Figure 2:**
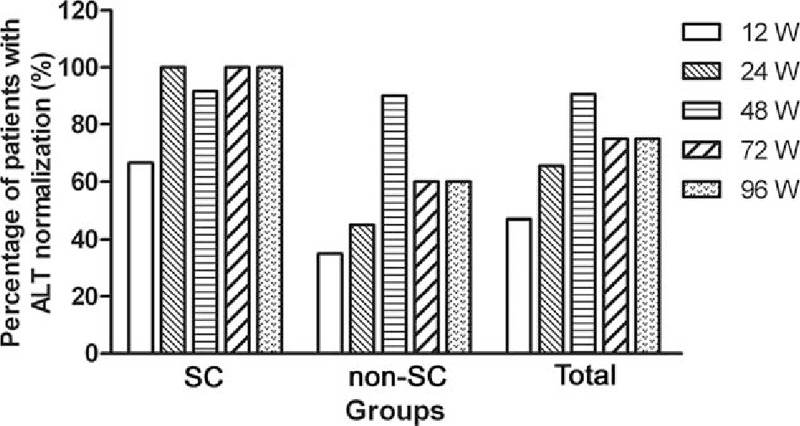
Number of patients with normalization of alanine transferase (≤48 U/L) during the 96 weeks treatment with tenofovir disoproxil fumarate. ALT = alanine transferase, TDF = tenofovir disoproxil fumarate.

The ALT, normalization rates were 66.7%, 100%, 91.7% 100%, and 100% at the end of 12, 24, 48, 72, and 96 weeks, respectively, for SC patients during TDF treatment. For non-SC patients, however, percentage of patients with ALT normalization during treatment were 35%, 45%, 90%, 60%, and 60% at the 5 time points, respectively (Fig. 2). The detail changing patterns of ALT level for each SC and non-SC patient over the course of 96 weeks of antiviral treatment period are shown (Fig. 1, Supplemental Digital Content 3).

### 3.3 Relationships between HBV DNA load or Treg frequency and ALT levels in SC and non-SC patients

**Figure 3:**
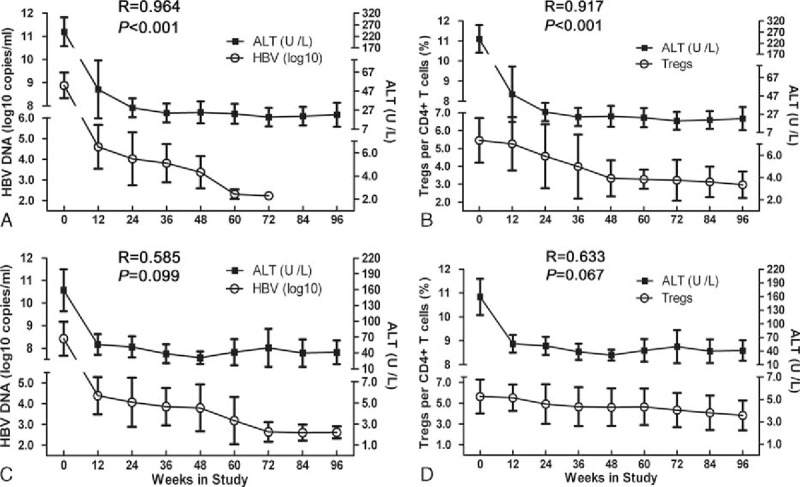
Association between hepatitis B virus (HBV) deoxyribonucleic acid (DNA) or regulatory T-cell (Treg) and alanine aminotransferase (ALT) level in hepatitis B e antigen seroconversion (SC) or non-SC patients during treatment relationships between the serum HBV DNA (log_10_ copies/mL) and ALT (U/L) levels are shown for SC (A) and non-SC (C) patients during tenofovir disoproxil fumarate (TDF) treatment. Relationships between the circulating CD4^+^CD25^+^ Treg frequencies (%) and serum ALT (U/L) levels are shown for SC (B) and non-SC (D) patients during TDF treatment. The X-axis indicates the different time points, the left Y-axis in (A) and (C) shows HBV DNA, the left Y-axis in (B) and (D) shows Treg frequencies, and the right Y-axis shows ALT levels. Correlations were analyzed using Spearman rank correlation analysis.

Throughout antiviral TDF treatment, if therapy yields SC, the HBV DNA load and ATL levels presented a significantly positive relationship (*R* = 0.964, *P* < 0.001) (Fig. 3A); the Treg frequencies and ALT declined were significantly correlated (*R* = 0.917, *P* < 0.001) (Fig. 3B) too. If not SC, then no correlations can be made (*R* = 0.585, *P* = 0.099; *R* = 0.633, *P* = 0.067) (Fig. 3C and D).

### 3.4 Predominantly skewed TRBVs used in SC and non-SC patients

The ratio of skewed TRBV families was compared for the SC and non-SC groups at 3 time points (baseline, week 72, and week 96) during the TDF treatment period. Six skewed (monoclonality or oligoclonality) TRBV families (BV3, BV11, BV12, BV14, BV20, and BV24) were more predominantly used than the others (TRBV families) in SC patients at baseline. Similarly, 5 TRBV families (BV5.1, BV11, BV12, BV20, and BV22) were used preferentially by the non-SC group at baseline (Table 3). Of these, 3 TRBV families (BV5.1, BV12, and BV22) were preferentially used in non-SC patients throughout the entire course of treatment (at baseline, week 72, and 96), but no TRBVs were prevalent in SC patients at weeks 72 and 96.

**Table 3:**
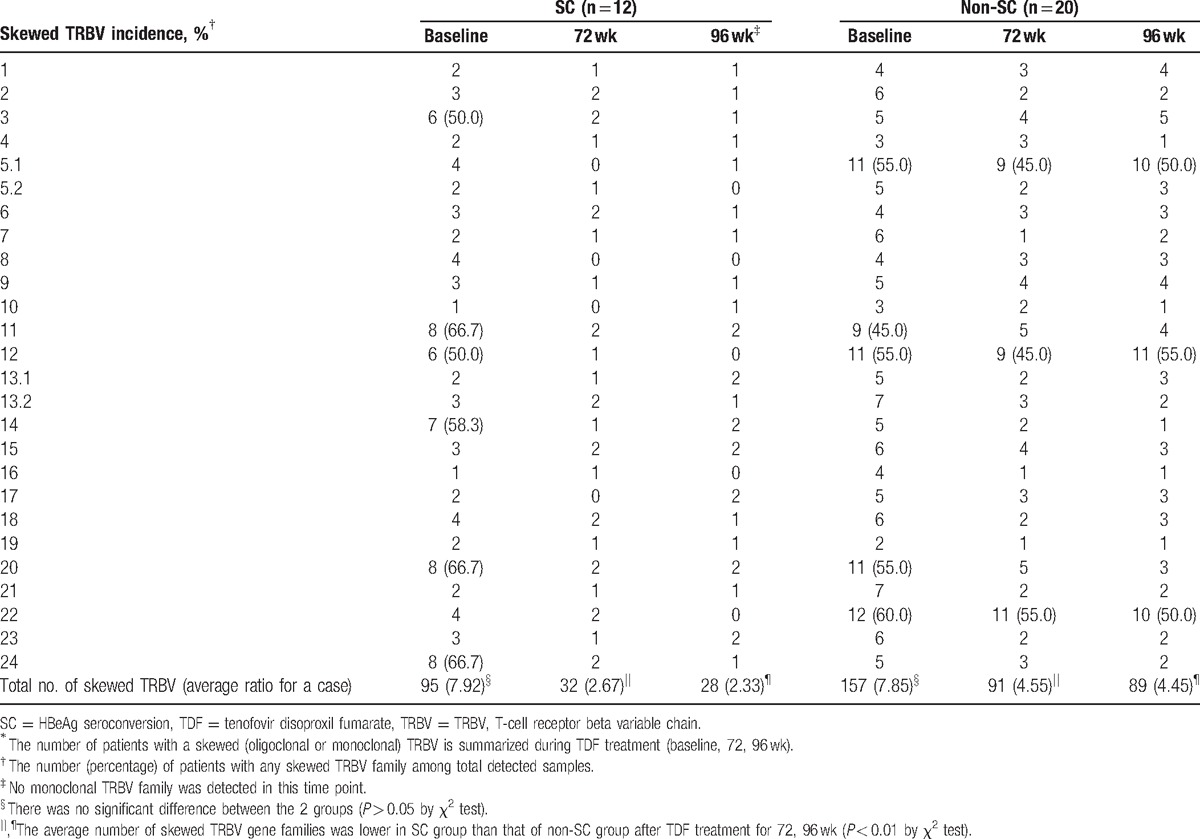
Frequency of skewed TRBVs in PBMCs from SC and non-SC patients during TDF treatment (baseline, week 72, and week 96)^∗^.

### 3.5 Conservative CDR3 motifs of TRBV in PBMCs from SC and non-SC patients

Table 4 shows representative CDR3 motifs in PBMCs from SC or non-SC patients during TDF treatment. In SC patients, the prevalent TRBV3 exhibited a relatively conserved CDR3 “XXXXX(X)-ETQ with BJ2.5 or BJ2.7;” CDR3 of TRBV14, BV24 primarily exhibited “XXX-NEQ with BJ2.1,” “XXXX-GSLN-XX with BJ2.1 or BJ2.5,” respectively; furthermore, the relative conserved TRBV20 was found only in SC patients and exhibited “XXXX-HSPL with BJ1.6.” However, in non-SC patients, TRBV5.1 and BV12 were used at a high frequency, but no relative conserved CDR3 motif was identified; additionally, CDR3 of TRBV22 was expressed as “XX(X)-REGL-XXX (XX) with BJ2.1” only in non-SC subjects. Although the TRBV11 was used predominantly in both in SC and non-SC patients at baseline, the CDR3 motif was different between the groups. In SC patients, the TRBV11 CDR3 expressed as “VATDEQ with BJ2.1,” but the “XXX-TGRA was used with BJ2.7” in non-SC patients.

**Table 4:**
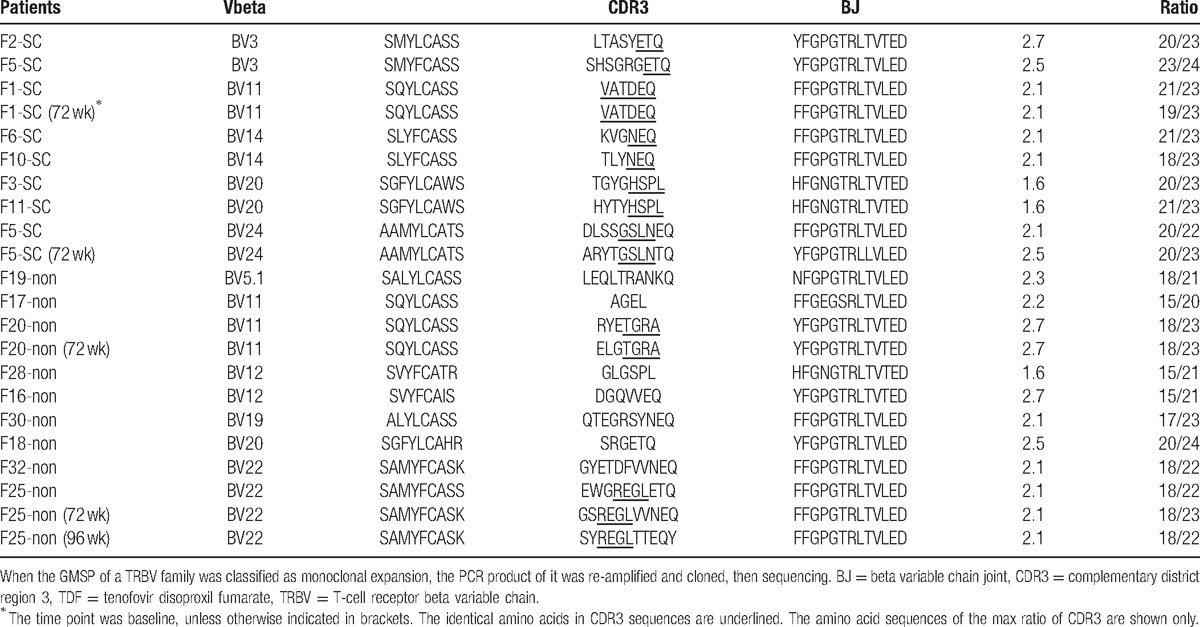
Representative amino acid sequences of monoclonal TRBV families in PBMCs from SC or non-SC patients during treatment.

## 4 Discussion

The present study provided a profile of select biochemical, virological, and immunological parameters in HBeAg-positive CHB patients treated with TDF for 96 weeks. Although HBsAg was not cleared by treatment, ALT and HBeAg SC correlated with successful therapy. For patients who did not seroconvert, the correlations of effective treatment were weaker. Specific TRBV families were also identified for HBeAg SC and non-SC CHB patients during TDF treatment.

Chronic HBV infection is a serious public health problem throughout the world, and 15 to 40% of CHB patients could develop into liver cirrhosis or HCC. Anti-HBV therapy has been recognized as an important way by which to reduce the incidence of HBV-related diseases.^[5,21]^ The antiviral treatment can inhibit and control HBV replication leading to loss of HBeAg expression in HBeAg-positive CHB patients. In addition, HBeAg SC is another indicator for a favorable outcome of HBeAg-positive CHB patients following anti-HBV treatment.^[22]^ In previous study, we discovered that loss of HBsAg detection could be used to predict HBeAg SC of CHB patients following TDF treatment^[19]^; moreover, interferon-inducible protein 10 levels could predict HBeAg SC in CHB patients with 5 years of entecavir treatment.^[23]^

Serum ALT levels are indicative of injury to liver cells and elevated serum ALT levels indicate the presence of active inflammation and hepatocyte injury caused by immune reactions against HBV. A higher ALT level can correlate with earlier HBeAg SC in CHB patients with lamivudine treatment.^[24]^ In this study, patients undergoing antiviral treatment who also SC for HBeAg developed normal serum ALT levels. Normal serum ALT levels indicate significant reduction in immunopathogenesis and liver damage.^[25,26]^

In contrast, HBeAg-positive CHB patients undergoing treatment who had normal ALT levels did not undergo SC and this correlated with poor efficacy of antiviral therapy. Tai et al^[27]^ reported that persistently normal ALT levels were associated with an excellent long-term prognosis of subjects with CHB infection. Our results agree and show that declining ALT levels were associated with a decrease in HBV DNA levels during antiviral therapy and that the significant positive relationship between them was determined only in SC patients, not in non-SC patients, which is consistent with the results of previous studies and further suggests that quantitative ALT levels indicate a positive prognosis for CHB patients undergoing antiviral treatment.^[28]^ The number of individuals achieving normalization of ALT levels increased over the 96 weeks of the trial with all SC patients achieving normalization but with only 60% for the non-SC patients. This suggests that the combination of these parameters be used as indicator of therapeutic efficacy.

The frequency of Tregs typically increases in CHB patients and is restored to normal levels in resolved CHB patients subjected to antiviral treatment.^[29]^ Tregs can be identified as CD4^+^CD25^+^ in HBV-infected patients.^[30,31]^ CD4^+^CD25^+^ Treg frequency did not significantly differ between the SC and non-SC patients before treatment (baseline) (Fig. 2, Supplemental Digital Content 4), but patients with SC had decreased frequency of Tregs. A reduction in Tregs may have allowed development of a more effective immune response that control infection and allow production SC for HBeAg.

HBV antigen-specific TRs are expressed on Tregs and cotytoxic T lymphocyte (CTLs). Prevalence of certain TRBV families associated with SC may represent those T cell receptors expressed on CTLs, whereas those TRBV families associated with non-SC patients or untreated CHB patients may represent receptors on Tregs. Further study of these TRBV families may help identify antigenic peptides for vaccines that can elicit helpful CTL responses rather than regulatory responses.^[32]^ Several skewed TRBV families (BV3, BV11, BV12, BV14, BV20, and BV24) that had relatively higher usage than other TRBV families in SC patients were identified in the present study, which will contribute to explore the relationship between these TRBV families and HBeAg SC in CHB patients.^[33]^ In addition, 3 TRBVs (BV5.1, BV12, and BV22) were preferentially used in non-SC patients during the treatment period, which suggested that these TRBV families be associated with the persistent HBV infection in non-SC patients. Therefore, these characteristic TRBVs and relative CDR3 motif may be relevant to HBeAg SC or non-SC of CHB patients during TDF treatment and could help to explore the TRBV repertoire (using high-throughput sequencing) in CHB patients with antiviral treatment.^[33,34]^

In summary, we found that quantitative serum ALT was associated with the HBeAg seroconversion of CHB patients during TDF treatment and indicated that ALT could be a potential predictive seromarker for distinguishing SC from non-SC patients, with the ALT cutoff value (41.5 U/mL) at week 24. Furthermore, determination of the preferentially used TRBV families, and their relatively conserved CDR3 motifs, that are associated with chronic progression of CHB provides an additional marker for monitoring effective antiviral therapy.^[35,36]^

## Supplementary Material

Supplemental Digital Content
