# Supplementary material for: Predictive value of serum ALT and T-cell receptor beta variable chain for HBeAg seroconversion in chronic hepatitis B patients during tenofovir treatment
Source: Medicine (Baltimore). 2017 Mar 10;96(10):e6242. doi: 10.1097/MD.0000000000006242 (PMC5348167; doi:10.1097/MD.0000000000006242)
Supplement: Supplemental Digital Content [file medi-96-e6242-s001.pdf]

Supplemental Digital Content 1

Table 1. Serum HBeAg (log10, S/CO) levels of 12 patients with SC during TDF treatment for 96 weeks

Patients	Base	12w	24w	36w	48w	60W	72w	84w	96w
**F1**	1.65	Non	Non	Non	Non	Non	Non	Non	Non
**F2**	3.01	0.85	0.35	0.08	Non	Non	Non	Non	Non
**F3**	3.05	1.80	1.40	1.18	Non	Non	Non	Non	Non
**F5**	2.79	2.06	1.53	2.22	0.99	1.11	Non	Non	Non
**F6**	2.69	1.00	Non	Non	Non	Non	Non	Non	Non
**F7**	2.39	Non	Non	Non	Non	Non	Non	Non	Non
**F9**	2.15	1.35	1.51	1.08	0.75	0.95	Non	Non	Non
**F17**	2.15	1.12	0.75	Non	Non	Non	Non	Non	Non
**F21**	3.15	1.81	1.23	1.16	0.98	Non	Non	Non	Non
**F25**	2.15	1.51	0.45	Non	Non	Non	Non	Non	Non
**F26**	2.71	0.78	0.71	1.15	0.39	0.47	Non	Non	Non
**F30**	3.15	2.01	1.77	1.25	Non	Non	Non	Non	Non

Non, non-reaction or the detection value (log10) <0; SC, HBeAg seroconversion; TDF, tenofovir disoproxil fumarate

Supplemental Digital Content 2

Table 2. Serum HBeAg (log10, S/CO) levels of 20 patients with non-SC during TDF treatment for 96 weeks

Patients	Base	12w	24w	36w	48w	60W	72w	84w	96w
**F4**	2.55	1.37	1.43	1.43	0.71	0.61	0.88	1.00	1.05
**F8**	2.20	0.75	0.37	0.63	0.22	0.21	0.29	0.00	0.12
**F10**	3.17	3.15	2.95	3.09	2.75	2.98	2.36	0.97	0.82
**F11**	2.60	1.84	1.35	1.25	1.12	1.22	1.25	1.15	1.07
**F12**	3.08	2.98	2.88	2.89	2.44	2.31	1.92	1.94	1.89
**F13**	3.15	0.47	0.84	1.08	1.17	1.24	0.87	0.71	1.25
**F14**	2.72	1.79	1.63	1.20	1.29	1.49	1.57	1.45	0.52
**F15**	3.11	2.15	2.15	2.05	1.82	1.97	1.81	1.21	1.11
**F16**	2.31	0.81	0.65	0.63	0.62	0.59	0.61	0.51	0.45
**F18**	3.15	2.97	2.78	2.69	2.39	2.55	2.31	2.01	1.85
**F19**	2.65	1.35	1.01	1.11	0.72	0.56	0.55	0.50	0.45
**F20**	2.21	0.91	0.55	3.01	0.61	2.12	2.12	1.85	1.68
**F22**	2.45	2.81	2.51	2.26	0.37	0.78	0.81	0.85	0.65
**F23**	3.02	1.51	0.81	0.12	1.91	1.56	1.15	1.22	1.57
**F24**	3.25	0.67	2.17	2.21	2.17	2.55	1.04	1.52	0.69
**F27**	2.78	2.22	0.52	1.12	2.28	0.98	1.36	0.56	0.85
**F28**	3.12	0.78	2.81	2.52	1.12	0.55	1.15	0.81	0.65
**F29**	2.41	2.21	1.25	1.25	1.21	1.15	1.12	0.85	0.55
**F31**	3.21	2.68	2.21	0.56	0.38	0.89	0.71	0.45	0.78
**F32**	2.61	1.91	1.78	1.15	1.55	1.23	1.12	0.85	0.41

non-SC, non HBeAg seroconversion; TDF, tenofovir disoproxil fumarate

Supplemental Digital Content 3

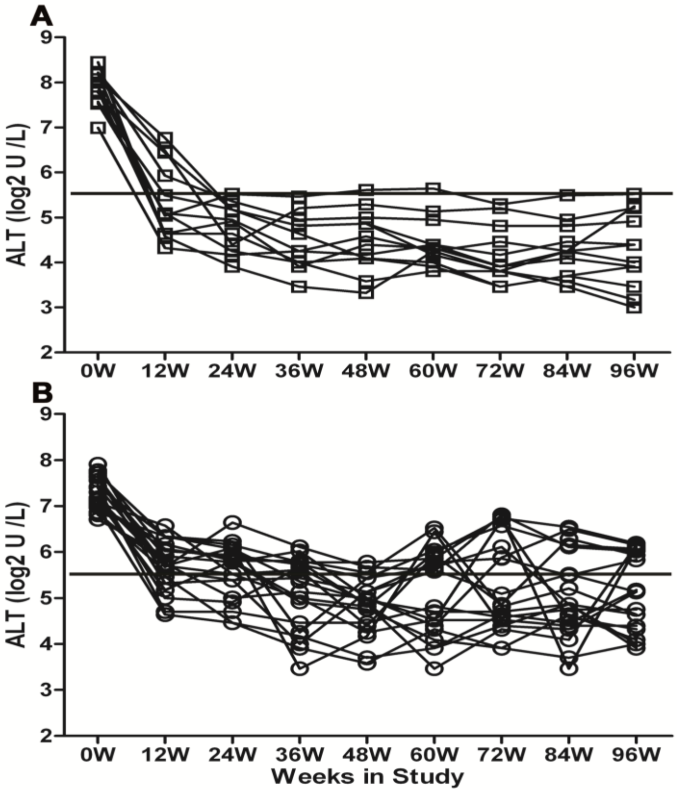

Figure 1. Changing profile of serum ALT (log2 U/L) levels of each patient was classified into SC (A) or non-SC (B) patients during TDF treatment for 96 weeks

Horizontal full line shows normal level of ALT (48 U/L, log2).

ALT, alanine transferase; non-SC, non HBeAg seroconversion; SC, HBeAg seroconversion; TDF, tenofovir disoproxil fumarate

Supplemental Digital Content 4

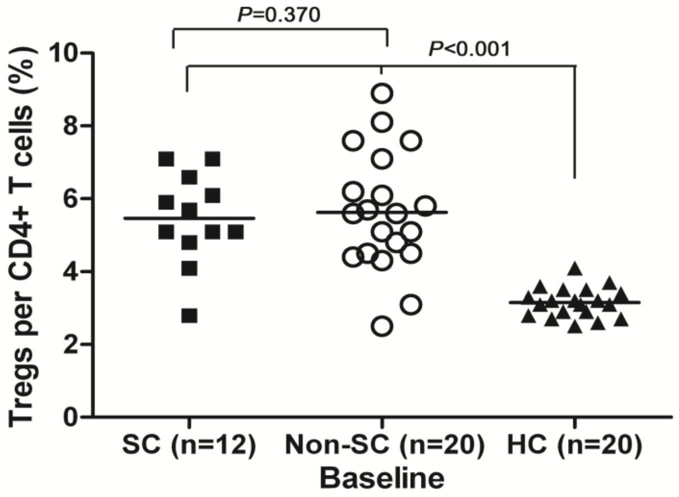

Figure 2. Circulating $CD4^+CD25^+$ regulatory T cells (Tregs) frequencies (%) in SC and non-SC patients before TDF treatment (baseline)

Data are expressed as a scatter diagram in which the midpoint of the black solid line is the mean Treg quantity.

HC, healthy control; non-SC, non HBeAg seroconversion; SC, HBeAg seroconversion; TDF, tenofovir disoproxil fumarate
